# Real-Time Monitoring of Bond Slip between GFRP Bar and Concrete Structure Using Piezoceramic Transducer-Enabled Active Sensing

**DOI:** 10.3390/s18082653

**Published:** 2018-08-13

**Authors:** Kai Xu, Changchun Ren, Qingshan Deng, Qingping Jin, Xuemin Chen

**Affiliations:** 1 College of Urban Construction, Wuhan University of Science and Technology, Wuhan 430065, China; xukai@wust.edu.cn (K.X.); renfighting@163.com (C.R.); shanadolph@163.com (Q.D.); 2 Department of Engineering, Texas Southern University, Houston, TX 77004, USA

**Keywords:** glass fiber-reinforced polymer (GFRP), bond slip, damage detection, piezoceramic transducers, wavelet packet analysis

## Abstract

Glass fiber-reinforced polymers (GFRPs) have received increasing attention in recent years due to their overall performance of light weight, low cost and corrosion resistance, and they are increasingly used as reinforcement in concrete structures. However, GFRP material has low elastic modulus and linear elastic properties compared with steel bars, which introduces different bonding characteristics between bars and concrete. Therefore, a reliable monitoring method is urgently needed to detect the bond slip in GFRP-reinforced concrete structures. In this paper, a piezoceramic-based active sensing approach is proposed and developed to find the debonding between a GFRP bar and the concrete structure. In the proposed method, we utilize PZT (lead zirconate titanate) as two transducers. One acts as an actuator which is buried in the concrete structure, and the other acts as a sensor which is attached to the GFRP bar by taking advantage of machinability of the GRRP material. Both transducers are strategically placed to face each other across from the interface between the GFRP bar and the concrete. The actuator provokes a stress wave that travels through the interface. Meanwhile, the PZT patch that is attached to the GFRP bar is used to detect the propagating stress wave. The bonding condition determines how difficult it is for the stress wave traveling through the interface. The occurrence of a bond slip leads to cracks between the bar and the concrete, which dramatically reduces the energy carried by the stress wave through the interface. In this research, two specimens equipped with the PZT transducers are fabricated, and pull-out tests are conducted. To analyze the active sensing data, we use wavelet packet analysis to compute the energy transferred to the sensing PZT patch throughout the process of debonding. Experimental results illustrate that the proposed method can accurately capture the bond slip between the GFRP bar and the concrete.

## 1. Introduction

Corrosion of steel reinforcement embedded in reinforced concrete (RC) is one of the most significant factors limiting the service life of RC structures [1,2]. It is even more severe in coastal and marine environments [3]. In particular, the use of chlorine salt in cold regions for snow removal results in chloride erosion of steel bars, which increases the maintenance costs of the structures [4]. A direct approach to resolve the corrosion problems would be to replace the steel reinforcement with fiber-reinforced polymer (FRP) bars in concrete structures [5]. Due to the extensive use of nonmetallic technology of reinforcements, several design guidelines have been published especially for concrete structures reinforced with FRP bars [6,7,8]. In recent years, glass fiber-reinforced polymer (GFRP) material has received increasing attention due to its light weight, low cost and corrosion resistance [9,10]. However, GFRP bars have low elastic modulus and linear elastic properties compared with steel bars, which introduces different bonding characteristics between bars and concrete [11]. Researchers have done some theoretical analysis and tried to build some models to describe the bonding conditions [12,13,14]. Apparently, the safety and durability of the concrete structure can only be assured when a safe bonding condition between concrete and GFRP bars is maintained. Studies show that interfacial debonding failure between GFRP bars and concrete may cause severe damage to the entire structure [15,16]. Therefore, a reliable monitoring system is urgently needed for debonding detection in GFRP-reinforced concrete structures.

There are many nondestructive testing (NDT) and structural health monitoring (SHM) [17,18,19] approaches to detect bonding damage in RC structures, including a radar technique, electromechanical impedance (EMI) method [20,21], impact-echo (IE) approach, ultrasonic surface waves (USW), among others. A considerable number of analytical and experimental investigations based on NDT have been published. Büyüköztürk and Yu developed an NDT radar with an airborne horn antenna for detecting near-surface debonding in GFRP-wrapped concrete columns [22]. Na and Baek took glass fiber composite plates as specimens and applied an EMI method to monitor adhesive debonding [23]. Azari et al. tested several detective concrete slabs of varying thickness using IE and USW methods [24]. The ultrasonic method also played a great role in detecting the damage in concrete structures [25,26,27]. Besides the methods mentioned above, there are some other techniques utilized in this field. Li et al. studied the structural debonding using a fiber-optical Doppler sensor [28]. Dérobert et al. focused on the assessment of cover concrete moisture content using ground penetrating radar (GPR) technique [29]. Also, the FEM (finite element method) is used for debonding study [30]. Since the bonding behavior between the reinforcing bars and the surrounding materials are important [31,32], pull-out tests are commonly used to experimentally study the bonding behavior between the reinforcing bar and the concrete [33].

Piezoceramic material is very versatile and is commonly used in SHM and damage detection [34,35,36,37,38]. Zhu et al. mounted piezoelectric components on reinforcing bars to detect the delamination between the steel bars and concrete in RC structures [39]. Kong et al. used piezoceramic-based smart aggregates (SAs) to monitor the very-early-age concrete hydration characterization [40]. Xu et al. embedded SAs in concrete of different strengths to study the progress of the formation of the different grades of the material [41]. Oliveira et al. developed an improved EMI method and neural networks to improve the efficiency of damage detection [42]. Wang et al. used a wearable piezoelectric device to monitor the pre-stress level of rock bolts [43]. Shao et al. applied a piezoelectric impedance-based method to detect bolt looseness in engineering structures [44]. Moreover, research has been performed to promote the piezoelectric sensing technique to practical applications. Baptista et al. investigated the effect of environmental temperature on the electrical impedance of lead zirconate titanate (PZT) sensors [45]. Perera et al. developed a flexible wireless smart sensor framework for structural health monitoring based on the EMI method and the self-sensing properties of the PZTs [46].

For its effectiveness and simplicity, the piezoceramic-based active sensing method has been proposed and applied for the debonding detection in recent years. Ihn and Chang used active sensing methods for detecting cracks and debonds in metallic and composite structures [47]. Mustapha et al. used a pair of PZTs to investigate the debonding in composite sandwich structures [48]. Providakis et al. used a PZT active sensing system to identify local damage in concrete structures [49]. Meng et al. fabricated an eccentric column and studied its damaging processes with the method of active sensing [50]. Xu et al. embedded SAs in the concrete core as actuators and bonded PZT patches on the outer surfaces of the specimen to detect the debonding between the steel tube and the confined concrete core [51]. Luo et al. developed a PZT-based ultrasonic active sensing method to monitor the compactness of concrete-filled FRP tubes with the help of the time-of-flight [52]. Feng et al. studied the detection of interfacial debonding in a rubber–steel-layered structure by using embedded piezoceramic transducers for active sensing [53]. Jiang et al. monitored the grouting quality of post-tensioning tendon ducts using a stress wave-based active sensing approach along with piezoceramic transducers [54].

The piezoceramic-based active sensing method has been used in the detection of the debonding status between concrete and the rebars [55,56]. However, there is no experimental approach to monitor the bond slip near the debonding area, which requires placing the transducers in the near-field. In this paper, a PZT-enabled active sensing technique is developed to monitor the bond slip between the GFRP bar and the concrete in a GFRP bar concrete structure. One PZT transducer is placed in the concrete, and one PZT patch is embedded in the GFRP bar because of the machinability of the GFRP. Both transducers are strategically placed across from the interface to efficiently monitor the interface condition via the active sensing approach. A bond slip between the GFRP bar and the concrete introduces a crack along the interface and adversely impacts the stress wave propagation across the interface since a pair of PZT transducers is used in this research to enable the active sensing. The PZT transducer in the concrete is used as an actuator to generate the stress wave that propagates across the interface, and the other PZT patch is used as a sensor to detect the arriving stress wave. In this paper, two specimens with the embedded PZT transducers are fabricated, and pull-out tests are performed on the two specimens. During the experiments, the active sensing data and the strain gauge measurements are recorded. To analyze the active sensing data, we use the wavelet packet analysis to calculate the energy associated with the stress wave. We expect a significant energy drop when the bond slip happens.

The rest of the paper is organized as follows. Section 2 introduces the basics about piezoceramic materials, and the principle of the active sensing-based bond slip detection method. Section 3 describes the experimental setup and procedures. Section 4 analyzes the experimental data and offers discussion. Finally, Section 5 concludes the paper.

## 2. Piezoceramic Transducer-Enabled Active Sensing

### 2.1. Basics of Piezoceramic Materials

Piezoceramic material, as a type of piezoelectric material, has superior properties of low cost, quick response, high reliability, solid-state actuation, wide frequency range and energy harvesting capacity [57,58,59]. A piezoelectric material will generate an electric charge when it is subjected to a stress or strain, and it will also produce stress or strain when an electric field is applied to it in its poled direction. For this special piezoelectric property, the piezoelectric material can be used as an actuator to generate a stress wave and as a sensor to detect a stress wave [60,61]. In this paper, PZT, which is a type of commonly used piezoceramic material with exceptional piezoelectric properties, is used.

### 2.2. Bond Slip Monitoring Using Piezoceramic Transducer-Enabled Active Sensing

An active sensing method [62,63,64] uses distributed transducers, which include at least two transducers: an actuator and a sensor. The actuator generates a stress wave, and the sensors measure the arriving signals. When a stress wave propagates through a region, the received signals, which carry the information of the propagation path, will reflect any changes in conditions or properties in the region.

**Figure 1:**
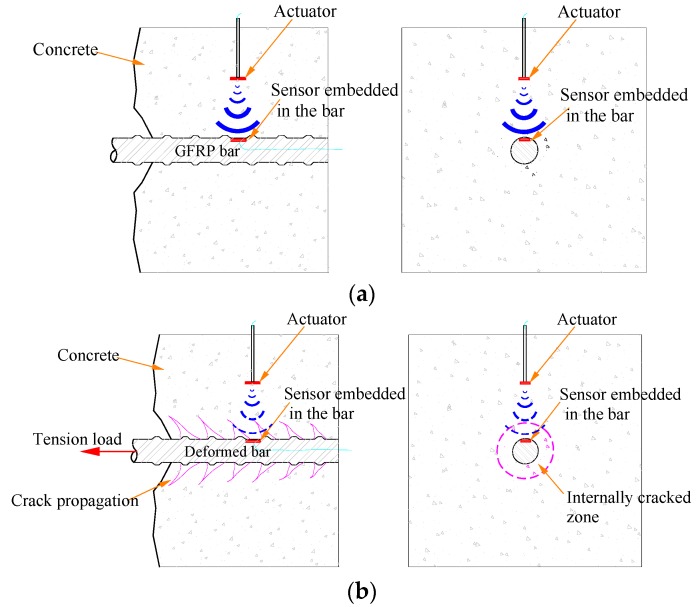
The principle of the active sensing approach detecting the debonding damage of GFRP bar–concrete structures: (**a**) health state (strong signal received by the SA sensors); (**b**) damaged state with debonding (weak signal received by the SA sensors).

In this paper, an active sensing technique using piezoceramic transducers is proposed to detect the debonding status of GFRP bars in RC structures. The PZT patch embedded in the GFRP bar is used as a sensor to detect stress waves which are generated from a PZT actuator fixed in the concrete specimen cube. The detecting principle is illustrated in Figure 1.

A pull-out test is used to cause the damage between the GFRP bar and the concrete specimen. When a bond slip happens, with the appearance of the internally cracked zone (shown in Figure 1b), which is an obstacle in the propagation path, the arriving stress wave will be attenuated by the process of the pulling-out test. The received signal that correlates with the degree of interfacial damage can be used to characterize the features of debonding of the GFRP bar and the concrete.

### 2.3. Wavelet Packet-Based Active Sensing Method

Wavelet packet analysis is an effective method for signal processing which has been widely used in structural health monitoring [65,66,67,68]. In the wavelet packet analysis, a signal is divided into an approximation and a detail. The approximation is then itself divided into a second-level approximation and detail to form the decomposition tree. Comparing to the traditional Fourier transform, the wavelet packet technique is a localized analysis in time-frequency of the signal. It enables the inspection of relatively narrow frequency bands over a relatively short time window.

Wavelet packet analysis is an excellent signal-processing tool which has been widely used to extract damage features. In this paper, we use Wavelet Toolbox provided by MATLAB to compute the total energy of the signal. The energy of each wavelet packet $E_{l}$ is computed based on the wavelet decomposition. The total energy of the signal is computed by the energy summation of all the wavelet packets which can be expressed as:

(1) $$E=\sum_{l=1}^{l=j}E_{l},$$

where *j* represents the decomposed wavelet packets. With the wavelet packet-based method, the severity of bond slip can be analyzed based on the total energy of the received signal.

## 3. Test Setup and Procedures

### 3.1. Specimen Design and Fabrication

**Figure 2:**
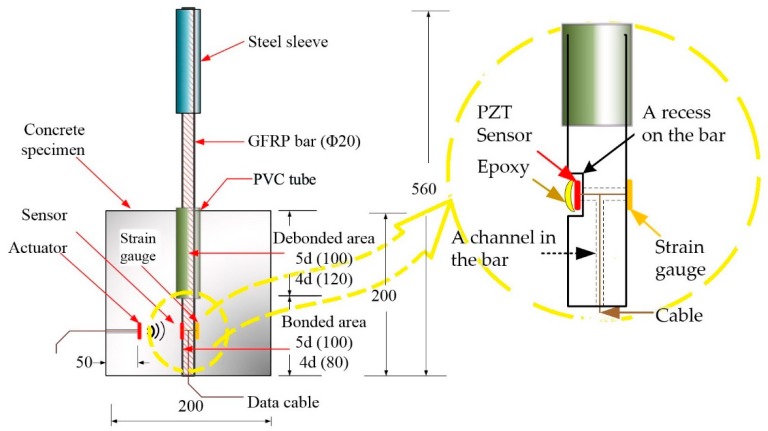
Schematic diagram of the specimens for the pull-out test (Unit: mm.)

The design of the specimen with the PZT-enabled active sensing approach is shown in Figure 2. The specimen includes mainly the concrete structure and the embedded GFRP bar. The GFRP bar has a steel sleeve for the ease of grip by a test machine. In addition, to ensure that the bond slip will be developed between the GFRP bar and concrete, a section of the bar in the concrete has a PVC tube. In this way, this section of the bar has no direct connection with the concrete, and only the bottom part of the bar is in direct connection with the concrete.

As shown in Figure 2, one PZT transducer is placed in the concrete as an actuator to generate stress waves, and one PZT patch 11 × 11 × 0.5 mm^3^ in size working as sensor is embedded in the GFRP bar by taking advantage of the machinability of the GFRP. With this special design, a recess that matches the dimension of the PZT patch is processed, and then the PZT patch is placed in the recess with a thin layer of epoxy. In this way, the original shape of the GFRP bar is restored. Both transducers are strategically placed across from the interface to best monitor the interface condition via the active sensing approach. A bond slip between the GFRP bar and the concrete introduces a crack along the interface and adversely impacts the stress wave propagation across the interface.

**Figure 3:**
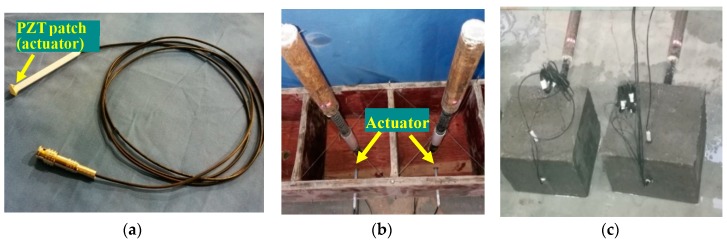
Fabrication of the test specimens. (**a**) A PZT patch actuator; (**b**) concrete mold; (**c**) concrete specimens.

The length and diameter of the GFRP bar are 560 mm and 20 mm, respectively, and the tensile strength and the modulus of elasticity of the GFRP bar are 83 MPa and 72 GPa, respectively. Each specimen has the dimensions of 200 mm × 200 mm × 200 mm. The strength of the concrete is 35 MPa. Two specimens are fabricated, and Figure 3 shows the fabrication of the test specimens. The actuator (Figure 3a) is placed in the concrete block, as shown in Figure 3b.

**Figure 4:**
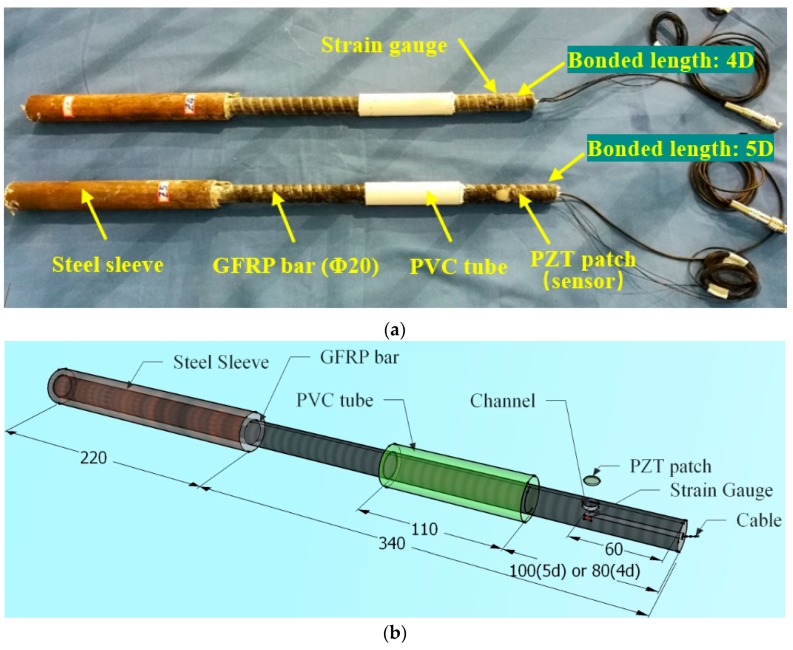
GFRP bars embedded with PZT patch sensor: (**a**) a photo of the GFRP bars used in the pull-out tests; (**b**) installation of the PZT sensor (Unit: mm.)

As shown in Figure 4, two GFRP bars are prepared for the two specimens. One has a bonded length of 100 mm, which is five times the diameter (labeled as 5D); while the other one has a bonded length of 80 mm, which is four times the diameter of the bar (labeled as 4D). Different bonded lengths mean different bonding areas, which leads to different carrying capacity and pull-out performance. The PZT sensor is embedded in the GFRP bars to get protection. To enable the embedment, we machine the GFRP bars with a shallow recess that matches the dimension of the PZT patch (Figure 2). With this design, the GFRP surface is still smooth, though part of it is the surface of the PZT patch. Meanwhile, a strain gauge is surface-bonded on the same section of the bar to monitor the local strain and to help to verify the experimental results of the PZT-based active sensing.

### 3.2. Mechanical Setup

**Figure 5:**
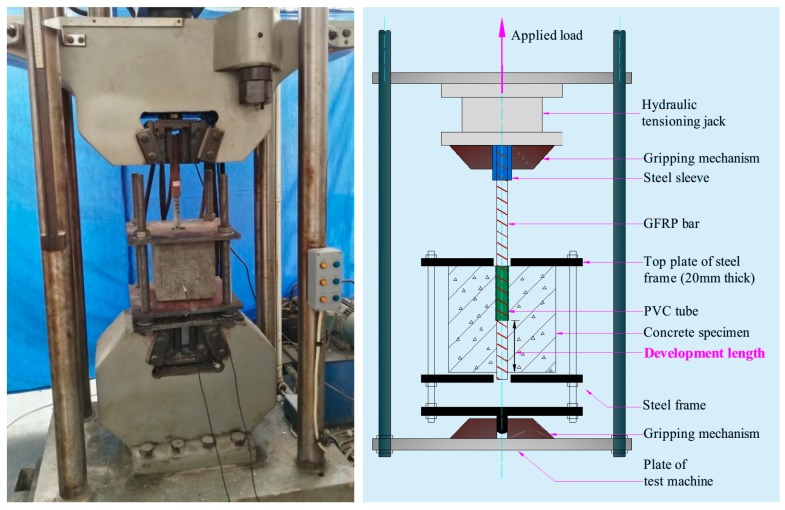
Loading equipment and steel loading frame.

The pull-out test schematic is shown in Figure 5. The concrete cube with the embedded GFRP bar is placed in a specially made steel frame that is positioned in the testing machine. The steel frame consisted of three 20-mm-thick steel plates, which are connected at the four edges with four rods 25 mm in diameter. The top plate has a hole in its center allowing the GFRP bar to run through. The steel sleeve on the GFRP bar is grabbed by the jaws of the gripping mechanism which is driven by a hydraulic tensioning jack. The bottom end of the steel frame is gripped in the jaws of the testing machine, which provides the reaction to the pull-out load.

### 3.3. Instrumental Setup

**Figure 6:**
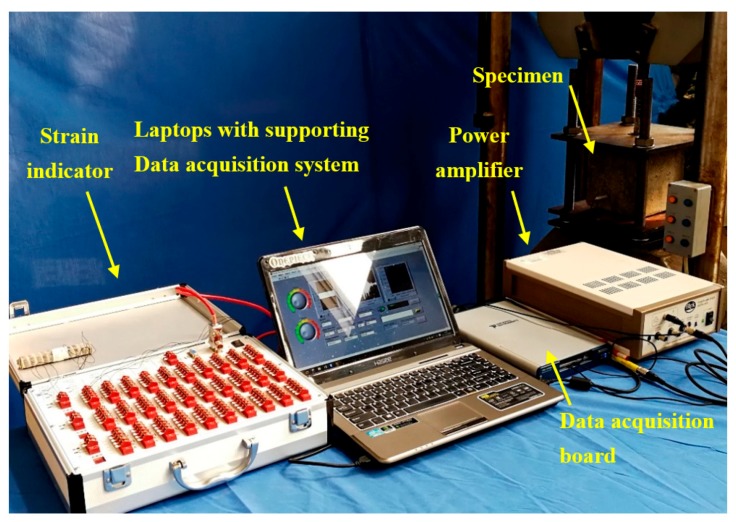
Instrument Setup.

The instrument system, as shown in Figure 6, includes the GFRP bar–concrete specimen, the NI data acquisition system (NI-USB 6361), and a laptop with NI LabVIEW, strain indicator, power amplifier and the universal material testing machine. A material testing machine with a tensile acquisition system is used to conduct the specimen pull-out test. The functionality of NI-USB 6361 is twofold: it has to generate the sine sweep excitation signal for the PZT patch actuator continuously, and secondly it has to collect the signal response from the PZT patch sensor embedded in the bar. The sweep sine signal frequency range is from 100 Hz to 250 kHz. The amplitude and period of the excitation signal are 10 V and 1 s, respectively.

### 3.4. Experimental Test Procedures

In this experimental test, the loads and the extensions applied on the specimen are controlled by the universal material test machine. Please note that in this research two experiments that involved two different specimens are conducted via pull-out tests. Both experiments are conducted under the displacement control mode. For both the strain gauge and PZT-enabled active sensing, the data are collected every 5 s. During both experiments, a bond slip is often accompanied by a loud sound. The experiment will be terminated if a sharp drop in the loading time history is observed.

**Experiment** **1.** *The specimen is the one with the 5D length of GFRP bar. The loading rate is 0.5 mm/min. The experiment lasted 18 min and 30 s*.

**Experiment** **2.** *The specimen is the one with the 4D length of GFRP bar. The loading rate is 0.3 mm/min. The experiment lasted 32 min and 5 s*.

**Figure 7:**
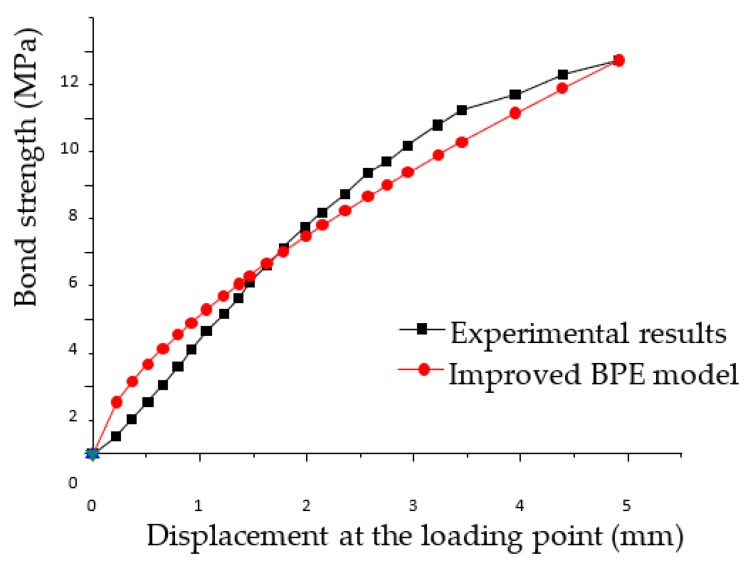
Comparison between the improved BPE model and the experimental results.

The BPE model, which is named after Bertero, Popov and Eligehausen, is generally used to describe the bond slip of a steel bar in concrete [69]. Cosenza et al. proposed an improved BPE model which can depict the bond slip curve of FRP bars in concrete [70], and the improved BPE model is more suitable to describe the bond slip occurring between FRP materials and the concrete. The improved model is verified by many experiments reported in [71,72]. In the research, we find that the experimental results show the same trend as the theoretical ones based on the improved BPE model (Figure 7). Indeed, the experimental and analytical results match very well.

## 4. Experimental Results and Discussion

**Figure 8:**
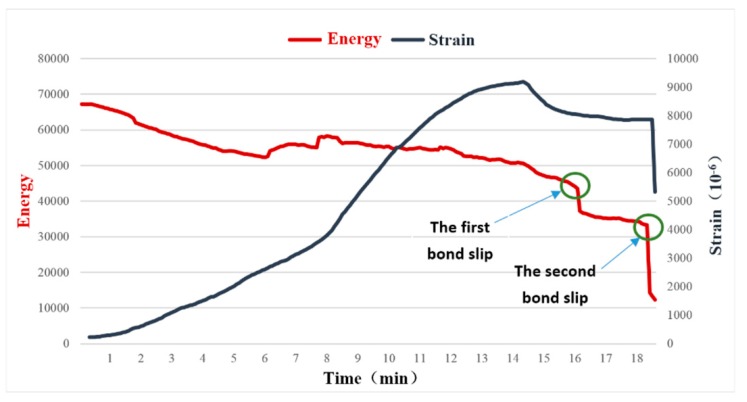
The energy and strain curves of the 5D specimen (Experiment 1).

**Figure 9:**
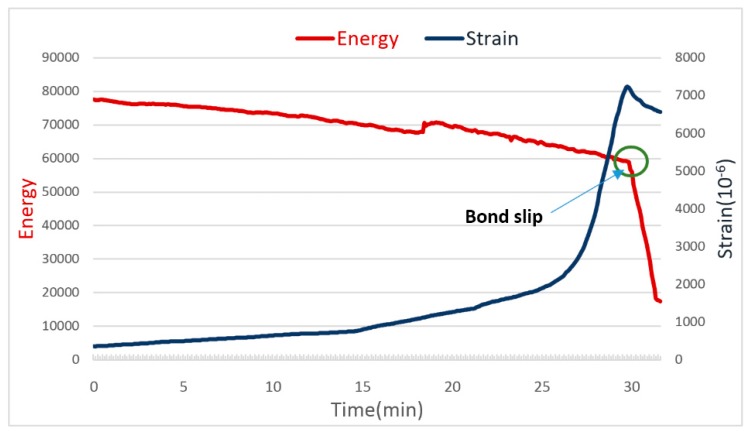
The energy and strain curves of the 4D specimen (Experiment 2).

We conduct both experiments by following the procedures described in Section 3. The first experimental results are shown in Figure 8, while Figure 9 shows the second experimental test results.

At the beginning of the loading process, the strain of the GFRP bar (black line) increases with the load until 14.3 min, after which the strain starts to decrease as shown in Figure 8. Please note the relationship between the strain and the load is highly nonlinear, which reflects the complex bonding between the GFRP bar and the concrete.

The strain gauge which is bonded on the surface of the GFRP through the epoxy is used to measure the local strain of the GFRP. The bonding condition changes during the loading process, which adds uncertainty to the strain monitoring. In addition, the strain gauge can only measure the strain in a small localized area. Therefore, it is difficult to effectively detect the bond slip between the GFRP bar and the concrete by using the strain value.

Also from Figure 8, the total energy of the stress wave received by the PZT sensor embedded in the GFRP bar, as represented by the red line, decreases with the applied load as a general trend. This reflects the fact that with an increase in the load, the bonding condition between the surfaces deteriorates, reducing the energy carried by the stress wave. The two sharp drops correspond to the two bond slips, as verified by the loud slipping sound during the experiment. It is worthwhile to point out that although the strain curve fails to reflect the first bond slip, which is a minor one, the active sensing method successfully detects this bond slip. For the second bond slip, which is a major one, both the strain gauge and the PZT transducers capture this event. The failed specimens are shown in Figure 9.

**Figure 10:**
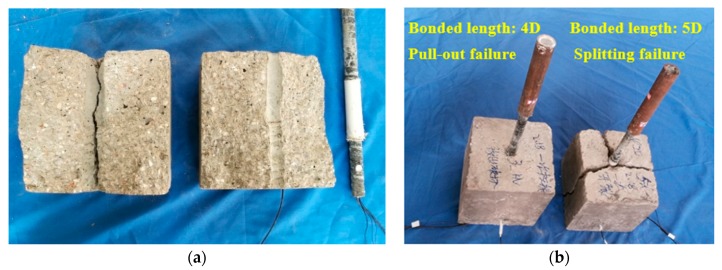
Different forms of failure: (**a**) the interface failure of the 5D specimen (splitting failure); (**b**) different forms of failure.

Similar results are also observed in experiment 2 with the second specimen. In this experiment, only a single major bond slip is observed, which is verified by both the energy reading of the PZT-enabled active sensing and the strain gauge. The failed specimen is shown in Figure 10. In summary, the PZT-enabled active sensing method successfully detects the bond slip between the GFRP and the concrete through pull-out tests in real time.

## 5. Conclusions

In this paper, a PZT-enabled active sensing technique was proposed and implemented to detect the bonding slip between a GFRP bar and RC structure. Two PZT transducers were employed: one in the form of a piezoceramic smart aggregate and the other in the form of a patch. Both transducers were strategically placed to face each other across from the interface between the GFRP bar and the concrete. A piezoceramic smart aggregate working as actuator was buried in the concrete to generate a stress wave that propagated and traveled through the interface. Meanwhile, the PZT patch that was embedded in the GFRP bar worked as sensor to detect the arriving stress wave. The bonding condition determines how the stress wave travels through the interface. The occurrence of a bond slip introduced a crack between the bar and the concrete, and dramatically reduced the energy the stress wave carried through the interface. In this research, two specimens were fabricated and two pull-out tests were conducted. The experimental pull-out test results demonstrated that the PZT-enabled active sensing approach could accurately capture the bond slip between a GFRP bar and concrete. The energy metric based on wavelet packet analysis can indicate the bond slip occurrence as the energy of arriving stress wave significantly drops. This active sensing technique provides a reliable real-time debonding damage monitoring method for GFRP-reinforced concrete structures.
